# Heat Shock Protein 90 (HSP90) and Her2 in Adenocarcinomas of the Esophagus

**DOI:** 10.3390/cancers6031382

**Published:** 2014-06-27

**Authors:** Julia Slotta-Huspenina, Karl-Friedrich Becker, Marcus Feith, Axel Walch, Rupert Langer

**Affiliations:** 1 Institute of Pathology, Technische Universität München, München 81765, Germany; E-Mails: slotta-huspenina@lrz.tum.de (J.S.-H.); kfbecker@lrz.tum.de (K.-F.B.); 2 Department of Surgery, Klinikum Rechts der Isar der Technischen Universität München, München 81622, Germany; E-Mail: marcus.feith@tum.de; 3 Institute of Pathology, Helmholtz Zentrum München, Deutsches Forschungszentrum für Gesundheit und Umwelt (GmbH), Neuherberg 85764, Germany; E-Mail: axel.walch@helmholtz-muenchen.de; 4 Institute of Pathology, University of Bern, Bern 3010, Switzerland

**Keywords:** HSP90, Her2, esophageal adenocarcinoma, immunohistochemistry, RPPA, *in situ* hybridization

## Abstract

Her2 overexpression and amplification can be found in a significant subset of esophageal adenocarcinomas. The activity of Her2 has been shown to be modulated by molecular chaperones such as HSP90. We analyzed expression/amplification data for HSP90 and Her2 on 127 primary resected esophageal adenocarcinomas in order to evaluate a possible relationship between these two molecules. HSP90 expression determined by immunohistochemistry was observed in various levels. Thirty nine (39) tumors (30.7%) were classified as Her2-positive according to their immunoreactivity and amplification status. There was a significant correlation between HSP90 expression and Her2-status (*p* = 0.008). This could also be demonstrated by quantitative protein expression analysis with reverse phase protein arrays (r = 0.9; *p* < 0.001). Her2-status was associated withpT-category (*p* = 0.041), lymph node metastases (*p* = 0.049) and tumor differentiation (*p* = 0.036) with a higher percentage of cases with negative Her2 status in lower tumor stagesA negative Her2-status was also associated with better survival in univariate and multivariate analysis (*p* = 0.001 and *p* = 0.014). For HSP90, no associations between clinical and pathological parameters were found. The observed association between HSP90 expression and Her2 suggests a co-regulation of these molecules in at least a subset of esophageal adenocarcinomas. Anti-HSP90 drugs, which recently have been introduced in cancer treatment, may also be an option for these tumors by targeting HSP90 alone or in combination with Her2.

## 1. Introduction

A significant percentage of adenocarcinomas of the upper gastrointestinal tract show overexpression and/or amplification of the membrane-bound tyrosin kinase and proto-oncogene Her2 (ERBB2). Since Her2 can be targeted by several drugs such as the monoclonal antibody trastuzumab this finding lead to the successful introduction of Her2 directed therapy in gastric cancer [1,2]. We and others have demonstrated that esophageal adenocarcinomas show Her2 positivity in a percentage comparable to or even higher than gastric cancer [3,4,5]. Her2 has been shown to interact with HSP90 (heat shock protein 90), a molecular chaperone belonging to the group of heat shock proteins [6]. These highly conserved molecules are responsible for the correct folding of other proteins, prevention of protein aggregation and protein activation [7]. Some data suggest that deregulated HSP90 expression may also support the effects of oncogenic Her2 [8] and this may represent a potential mechanism of resistance to Her2 directed drugs. On the other hand, inhibition of HSP90 may potentiate the effects of anti-cancer drugs targeting client proteins of this molecule [9,10,11,12,13]. 

The relationship between HSP90 and Her2 has not been investigated for esophageal adenocarcinomas so far. We have studied the role of Her2 and recently HSP90—among other molecular chaperones—in esophageal adenocarcinomas: overexpression and/or amplification of Her2 were associated with a more aggressive biological behavior in a well characterized collection of primary resected tumors. Similar expression profiles of molecular chaperones (heat-shock proteins and glucose-regulated proteins) were associated with patients’ prognosis in primary resected tumors and response to preoperative treatment in patients treated with neoadjuvant chemotherapy before surgery [3,14,15,16,17]. For the purpose of this correlative and descriptive study we analyzed the raw data of these previous tissue based studies, supplemented by some additional expression analysis, in order to evaluate a possible association and co-regulation of these molecules.

## 2. Experimental

### 2.1. Patients and Tissues

The case collection consisted of 127 formalin fixed, paraffin embedded (FFPE) archival cancer tissue from patients with esophageal adencarcinomas who underwent primary surgical resection (trans-thoracic or trans-hiatal esophagectomy) between 1993 and 2005 at the Klinikum Rechts der Isar of the Technische Universität München (Germany). The resection specimens were processed immediately after surgery, *i.e.*, they were opened by a pathologist and fixed in 4.5% buffered formalin for 24–48 h. Informed consent from all patients for the use of additional molecular analysis was given at the time of surgery. The use of archival tissue for molecular analysis was approved by the local ethical commission (No. 2136/08). The median age of the patients was 62 (range 33–82). Median overall survival (OS) was 72 months (95% CI = 34–110). All tumors were reclassified according to the current UICC TNM-classification [18]. Tumor grading was done according to the WHO Classification of Tumors of the Digestive System [19]. The pathologic characterization of the collective is given in Table 1.

**Table 1:** Characterization of the case collective of primary resected esophageal adenocarcinomas.

Parameter		n	%
pT category	pT1	57	44.9
	pT2	24	18.9
	pT3	46	36.2
Lymph node metastases	absent	76	59.8
	present	51	40.2
Distant metastases	absent	118	92.9
	present	9	7.1
Grading	G1	11	8.7
	G2	57	44.9
	G3	59	46.5
Resection status	R0	109	85.8
	R1	18	14.2
	total	127	

### 2.2. Immunohistochemistry

Immunohistochemistry for Her2 (clone 4B5, Ventana Medical Systems, Inc., Tucson, AZ, USA) and HSP90 (Abcam, Cambridge, UK) was performed on formalin-fixed and paraffin-embedded (FFPE) tissue on a tissue microarray (TMA) as described before [3,16]. 

**Figure 1:**
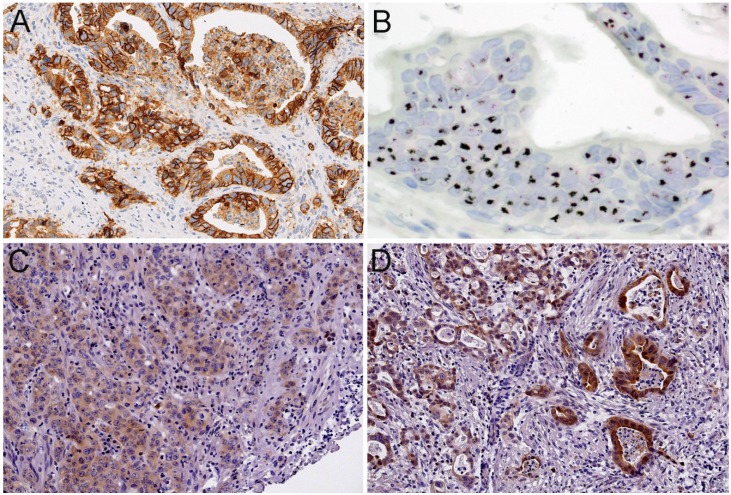
Examples of immunohistochemical stainings and in-situ hybridization: (**A**) Her2 immunohistochemistry 3+ (positive) ×20; (**B**) Her2 high level amplification by silver *in situ* hybridization/SISH ×40; (**C**) HSP90 immunohistochemical low expression ×20; (**D**) HSP90 immunohistochemical high expression; ×20.

Her2 immunoreactivity was assessed according to recommendations for Her2 testing in gastric carcinoma [20]: immunohistochemistry 3+ staining (Figure 1A) was defined as strong complete baso-lateral or lateral membranous staining of cells, irrespective of the percentage of tumor cells, visible at low magnification (×2.5–5), immunohistochemistry 2+ as weak to moderate baso-lateral or lateral membranous staining visible at ×10–20 magnification, and immunohistochemistry 1+ as weak membranous staining visible only with ×40 magnification. The criteria for Her2 assessment were used according to the proposal for the application on bioptic tissue [20].

The expression of HSP90 (Figure 1C,D) was determined based on the intensity of cytoplasmic staining and the percentage of stained tumor cells. Multiplication of scores for intensity of cytoplasmic staining and the percentage of stained cells resulted in an immunoreactivity score (IRS). A classification into negative—low—high expression was done according to the terciles of the distribution of IRS [16].

### 2.3. In Situ Hybridization and Definition of Her2 Status

Data for Her2 amplification were obtained from fluorescence *in situ* hybridization (FISH) or silver *in situ* hybridization (SISH) analysis [3,21]. A positive Her2 status was defined as immunohistochemical 3+ and/or amplification determined by ISH with a Her2/cep17 quotient ≥2 (Figure 1B). 

### 2.4. Protein Extraction, Reverse Phase Protein Arrays and Quantitative Expression Analysis

For 71 cases additionally quantitative protein expression data generated from reverse phase protein array (RPPA) analysis could be included. A detailed description of this approach has been given in previous publications [14,16,22]. In brief, immunoreactive protein was extracted from freshly cut sections of FFPE tissue, which then were processed in 100 µL of extraction buffer EXB Plus according to the supplier’s recommendations (Qproteome FFPE Tissue Kit, Qiagen, Hilden, Germany). Protein concentrations were determined using the Bradford protein assay according to the manufacturer’s instructions (BioRad, Hercules, CA, USA). Probing for β-actin by western blot was done in order to verify the success of the protein extraction and the suitability of the material for later reverse phase protein array (RPPA) analysis. 

RPPAs were generated using the Calligrapher MiniArrayer (BioRad) in accordance with the manufacturer’s instructions. Three replicates per lysate were applied in various dilutions to a nitrocellulose-coated glass slide (Grace Bio-Labs, Bend, OR, USA) in order to obtain a total of 18 data points per sample. Peroxidase blocking was performed according to the manufacturer’s instructions (Dako, Glostrup, Denmark). Immunodetection was performed similar to a western blot (antibodies: HSP90: Abcam, Cambridge, UK; Her2: Dako; pHer2^Tyr1248^: Invitrogen, Carlsbad, CA, USA). In parallel, staining with Sypro Ruby Protein Blot Stain (Molecular Probes, Eugene, OR, USA) was done for the estimation of the total protein amount. The TIFF images for the antibody-stained slides and Sypro Ruby-stained slides were analysed with MicroVigene 3.5.0.0 software (VigeneTech, Carlisle, MA, USA). The MicroVigene signal-intensity points (MVS) were calculated by the integral of a logistic four-point fit model, matched optimally to the 18 data points that had been obtained.

### 2.5. Statistical Analysis

IBM SPSS 21.0 Statistics statistical software (SPSS Inc., Chicago, IL, USA) was used for statistical analysis. Associations between expression and amplification data and pathological features were given in crosstabs and were evaluated with X^2^ test. For survival analysis, Kaplan-Meier estimates, log rank tests and Cox’s proportional hazards regression analysis were used. All tests were 2-sided, and the significance level was set at 0.05.

## 3. Results

### 3.1. HER2 Immunohistochemistry and in Situ Hybridization

Seventy four (74) tumors (58.3%) showed no Her2 immunoreactivity, 25 tumors (19.7%) had an immunoscore of 1+, 13 cases (10.2%) showed 2+ immunoreactivity and 15 cases (11.8%) 3+. Using the criteria mentioned above, which included *in-situ* hybridization of all tumors, 88 tumors (69.3%) were classified as Her2 negative, and 39 tumors (30.7%) as Her2 positive. 

### 3.2. HSP90 Immunohistochemistry

In 61 cases (48.0%) there was no immunoreactivity for HSP90. Forty four (44) tumors (34.6%) showed low HSP90 expression (IRS score 1–4) and 22 tumors (17.3%) showed high HSP90 expression (IRS score > 4).

### 3.3. Quantitative Protein Expression (RPPA)

Median quantitative expression of Her2 (Her2/SyproRuby) was 675 (range; 188–9058) and of pHer2 (pHer2/SyproRuby) was 726 (range; 161–3100). Median quantitative HSP90 expression (HSP90/Sypro Ruby) was 775 (range; 207–3245).

### 3.4. Association between Her2 and HSP90

**Figure 2:**
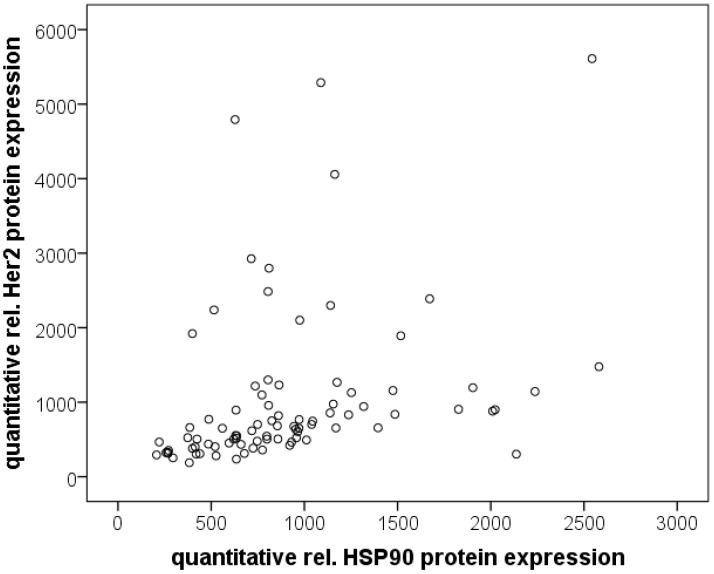
Scatterplot with RPPA data for quantitative relative Her2 expression and HSP90 expression.

There was a correlation between HSP90 expression assessed by immunohistochemistry and Her2 status (*p* = 0.008; Table 2): the rate of Her2 positive tumors in relation to HSP90 expression was 28% for HSP90 negative tumors. 69% of Her2 tumors showed HSP90 expression (44% HSP90 low tumors and 28% HSP90 high tumors). In contrast, the percentage of Her2 negative tumors in relation to HSP90 expression was decreasing from 57% (HSP90 negative tumors), over 30% (HSP90 low tumors) to 13% (HSP90 high tumors). The association between HSP90 and Her2 expression could also be demonstrated by quantitative RPPA analysis: Her2 *vs.* HSP90: Spearman-rho = 0.574; pHer2 *vs.* HSP90: Spearman-rho = 0.791 (*p* < 0.001 each; Figure 2). Her2 and pHer2 expression levels showed a highly significant correlation as well (Spearman-rho = 0.531; *p* ≤ 0.001). Interestingly, tumors with HSP90 immunoreactivity had slightly, but not significant higher Her2 levels by RPPA (*p* = 0.059), but not HSP90 or pHer2 levels (*p* = 0.432 and *p* = 0.486). Between Her2 status and RPPA expression data, no correlations were found.

**Table 2:** Correlation between HSP90 expression and Her2 status.

		Her2 Status		Total	*p*-value
		Negative	Positive		
HSP90 expression	negative	50	11	61	0.008
	low	27	17	44	
	high	11	11	22	
Total		88	39	127	

### 3.5. Clinicopathological Parameters and Survival Analysis

**Figure 3:**
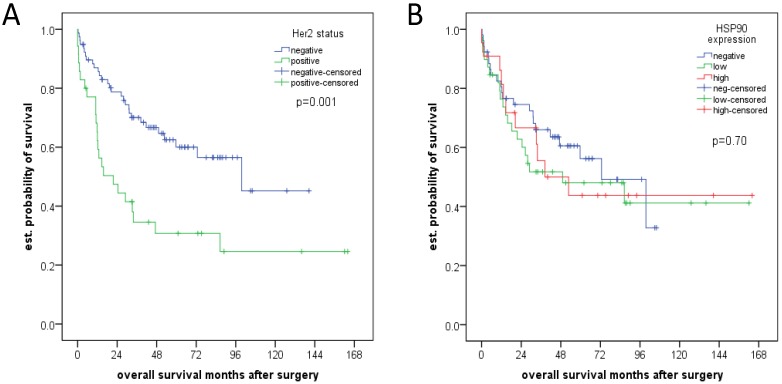
(**A**) Her2 status and overall survival; (**B**) HSP90 expression and overall survival.

There were no significant associations between pathologic features (pT, pN category, grading) and HSP90 (Table 3). Her2 positivity was associated with pT category (*p* = 0.041), lymph node metastases (*p* = 0.049) and tumor differentiation (*p* = 0.036; Table 4), in particular the percentage of Her2 negative tumors was higher in tumors with lower pT-category and without lymph node metastases as compared to Her2 positive tumors. Moreover, there were no well differentiated tumors (G1) with Her2 positivity. A negative Her2 status was also associated with better survival in univariate (*p* = 0.001; Figure 3A) and multivariate analysis (*p* = 0.005). This was not the case for HSP90 (Figure 3B; Table 5).

**Table 3:** Correlation between HSP90 expression and pathologic characteristics.

Parameter		HSP90 Expression			*p*-value
		Negative	Low	High	
pT category	pT1	30	20	7	0.232
	pT2	9	7	8	
	pT3	22	17	7	
lymph node mets.	absent	40	23	13	0.389
	present	21	21	9	
distant mets.	absent	57	41	20	0.921
	present	4	3	2	
grading	G1	8	3	0	0.246
	G2	29	17	11	
	G3	24	24	11	
total	127	61	44	22	

**Table 4:** Correlation between Her2 status and pathologic characteristics.

Parameter		Her2 Status		*p*-value
		Negative	Positive	
pT category	pT1	46	11	0.041
	pT2	14	10	
	pT3	28	18	
lymph node metastases	absent	58	18	0.049
	present	30	21	
distant metastases	absent	82	36	1.0
	present	6	3	
grading	G1	11	0	0.036
	G2	35	22	
	G3	42	17	
total	127	88	39	

**Table 5:** Multivariate analysis of prognostic relevant factors.

Factor	Exp(B)	95% CI for Exp(B)		*p*-value
		Min	Max	
pTcategory	1.292	0.824	2.025	0.264
lymph node mets	2.235	1.07	4.67	0.032
distant mets	1.69	0.711	4.016	0.235
grading	1.218	0.736	2.016	0.443
resection status	3.172	1.586	6.34	0.001
Her2 status	2.028	1.152	3.57	0.014

## 4. Discussion

The results of our analyses demonstrate an association between the expression of HSP90 and Her2 amplification/expression in esophageal adenocarcinomas which may present at least in a subset of tumors. This could be observed by immunohistochemistry and by quantitative protein expression analysis with reverse phase protein arrays (RPPA). In contrast to Her2, where an association between tumoral Her2 positivity and unfavorable clinical course had been reported before [3], no correlation between HSP90 alone and clinical and pathological parameters was found.

The rate of Her2 overexpressing or amplified esophageal adenocarcinomas [23] is comparable to gastric [2,24] and breast cancer [25]. In contrast to breast cancer, data about the prognostic impact of Her2 in gastrointestinal tumors are not conclusive. Although only investigated in retrospective analyses, Her2 positivity is presumed to be predictive for anti Her2-directed therapy, which recently successfully has been introduced in the treatment of metastasized gastric and gastro-esophageal adenocarcinomas [1]. The activity of Her2 has been shown to be influenced and partly controlled by so called “molecular chaperones”. These molecules assure accurate folding of other proteins thereby assisting in the maintenance of cellular integrity and homeostasis [26,27]. Heat shock proteins (HSPs) which are subdivided into several classes according to their molecular weight act as classical chaperones under normal conditions. Interestingly, deregulated activity of HSPs has been found in cancer cells. This may be caused by intrinsic malignant deregulatory effects but also by the altered interaction with other oncogenic molecules [6,28]. HSP90 is one of the most abundant cellular HSPs. 

Molecules such as Her2, EGFR, VEGFR, ALK, AKT, p52, cyclin D1, Met, Cdk4, HIF-1α or MMP2 which play important roles as oncogenes and are involved in essential pathways of cancer are described to be client proteins of HSP90 (for review [10,13]). 

This association caused interest on the impact HSP90 as potential anti-tumoral target. Inhibition of HSP90 may represent a combinatorial attack on multiple oncogenic pathways. In the last years, the development of anti-HSP90 directed drugs and inhibitors has rapidly moved forward. Geldanamycin derivatives (e.g., 17-AAG) were the first substances that showed that HSP90 is a druggable target for cancer therapy. Second generation and synthetic HSP90 inhibitors have improved pharmacological properties and safety profiles [13,29]. Anti-HSP90 therapy may be applied alone or in combination with other cytotoxic or directed therapies. Although up to date no agent has been approved for use in the clinical practice, targeting HSP90 is considered as exciting and promising new treatment approach and HSP90 inhibitors have been tested in many preclinical and clinical studies. A detailed description of these various studies would be beyond the scope of this paper, so we would like to refer to recent reviews and meta-analyses [10,13]. In brief, besides in ALK-rearranged NSCLC the highest efficacy for HSP90 inhibition so far has been reported for Her2-positive breast cancer: for example, HSP90 inhibition resulted in a dose-dependent and complete degradation of active (phosphorylated) HER2 and EGFR in Her2 overexpressing cell lines [30]. A novel HSP90 inhibitor, CH5164840 showed significant antitumor efficacy against gastric and breast cancer models which was even more enhanced when combined with HER2-targeting agents [31]. A combination therapy with the HSP90 inhibitor 17-AAG and trastuzumab showed significant anticancer activity in patients with HER2-positive metastatic breast cancer progressing on trastuzumab in a phase II study [32]. Basing on such results, HSP90 targeting alone or in combination with Her2 directed therapy is regarded as a promising novel approach for the treatment of breast cancer, which also may be a potentially successful way to overcome acquired or intrinsic trastuzumab resistance. Moreover, this may also be transferred onto other Her2 positive tumors, such as the subset of gastrointestinal adenocarcinomas with Her2 overexpression or amplification. On the tissue level, however, there are only scarce data about HSP90 and Her2 in human malignancies. Recently, we have reported a strong correlation between HSP90 and Her2 expression in gastric carcinomas [33] and colon carcinomas [34] which is in line with the observations of the present study. To our knowledge, other tissue based studies about the association of these molecules in other tumor types, however, are not available yet.

## 5. Conclusions

The results of our analyses may contribute to the learning about the biology of HSP90 and its client proteins, especially supporting an association between Her2 and HSP90 expression at least in a subset of cases on the tissue level. They warrant further functional investigation about the interaction of HSP90 and Her2 in gastrointestinal carcinomas both *in vitro* and *in-* or *ex vivo*. Basing on these data, the rationale for HSP90 as a potential targetable molecule may offer interesting alternative therapeutic options for gastrointestinal tumors, especially for Her2 positive gastric and esophageal adenocarcinomas [29,35]. 
